# Emergence of Skilled Behaviors in Professional, Amateur and Junior Cricket Batsmen During a Representative Training Scenario

**DOI:** 10.3389/fpsyg.2018.02012

**Published:** 2018-10-30

**Authors:** Jonathan D. Connor, Damian Farrow, Ian Renshaw

**Affiliations:** ^1^Institute for Health and Sport, Victoria University, Melbourne, VIC, Australia; ^2^National Cricket Centre, Cricket Australia, Brisbane, QLD, Australia; ^3^Movement Science Department, Australian Institute of Sport, Canberra, ACT, Australia; ^4^School of Exercise and Nutrition Sciences, Queensland University of Technology, Brisbane, QLD, Australia

**Keywords:** representative learning design, cricket batting, emergent behavior, expertise development, skill acquisition, game based learning, perception–action coupling

## Abstract

The aim of this study was to explore the emergence of skilled behaviors, in the form of actions, cognitions and emotions, between professional state level cricket batters and their lesser skilled counterparts. Twenty-two male cricket batsmen (*n* = 6 state level; *n* = 8 amateur grade club level, *n* = 8 junior state representative level) participated in a game scenario training session against right arm pace bowlers (*n* = 6 amateur senior club). The batsmen were tasked with scoring as many runs as possible during a simulated limited-overs game. The actions, cognitions, and emotions of each batsmen were recorded *in situ* with findings showing differences between state level players and those lesser skilled. State level batsmen played more scoring shots and scored more runs, underpinned by superior bat–ball contact and technical efficiency. Furthermore, the state player’s cognitive evaluations of their own performance differed from junior batters, with more reported strategies based on an external outcome focus, such as where to score runs, rather than a focus on internal processes, such as making technical changes. State level batsmen also reported lower levels of nervousness compared with junior level batsmen. These results highlight the importance of viewing the emergence of skilled behavior as multi-faceted, rather than simply the acquisition of superior execution and technical proficiency.

## Introduction

Analyzing skilful behaviors in sport performance has long been of great interest to researchers and practitioners alike. Unlike being exposed to a novel or unfamiliar stimulus, observing individuals with various levels of skill or prior experience within a sporting task can reveal crucial information about skilful behavior. Earlier experimental work typically followed a more reductionist approach, which allowed for highly standardized and controlled experiments that limited the number of variables influencing behavior (Hoffman, 1990; Singer, 1990). However, to better understand skilful behavior in more dynamic environments, there have been calls to progress toward methodological approaches that are more representative of the performance environment (Abernethy et al., 1994; Renshaw and Gorman, 2015). As such, skilful behavior can be viewed as the resultant product of an individual’s adaptive actions, cognitions and emotions to the evolving (i.e., dynamic) constraints in their environment. Testing environments, therefore, must contain key information that enables fidelity in the actions, cognitions and emotions of the performer attempting to achieve a specific performance goal (Pinder et al., 2011b; Seifert et al., 2013).

Measuring expertise in dynamic performance environments presents a complex challenge for researchers. Interceptive timing tasks, such as those occurring in fast ball sports, are commonly utilized as effective task vehicles in laboratory settings. Cricket batting, as an exemplar dynamic interceptive timing task, involves batters’ facing an opposition bowler and accompanying fielders whose intent is to ‘dismiss’ them for as few runs as possible. Differences between skilled and lesser skilled performers have been found in coordinative movements (i.e., biomechanics; Elliott et al., 1993; Stretch et al., 1995, 1998; Taliep et al., 2007; Penn and Spratford, 2012), pattern recognition of opposition kinematics (Müller et al., 2006; Renshaw et al., 2007; Müller and Abernethy, 2012) and spatio-temporal interceptive abilities (Weissensteiner et al., 2011). However, examining key processes underpinning expertise conducted outside the performance environment have been criticized for not being representative of the inherent complexity within dynamic tasks. In particular, there is a lack of research into the requisite adaptive behaviors that occur in response to the task goal, opposition actions, and performance environment (Araújo et al., 2007; Pinder et al., 2011b).

Recently, proponents of ecological dynamics have suggested that the concept of ‘representative task design’ should be considered when designing skill tests in sport (Vilar et al., 2012). Representative design refers to the degree in which conditions of the experiment represent the behavioral setting in which it is intended to exemplify (Brunswik, 1956; Araújo et al., 2007). In essence, representative task design highlights the need to ‘sample’ performance environments and ensure any tests of expertise are predicated on the key information sources found in such performance contexts (Brunswik, 1956). The importance of this need is evident from experiments breaking information–movement couplings led to a degradation and false account of the performance of experts (Oudejans et al., 1997; van der Kamp et al., 2008; Mann et al., 2010). Goodale and Milner (1992) proposed two, separate yet integrated, visual pathways for interceptive actions, that enables skilled performers to functionally adapt their behaviors. van der Kamp et al. (2008) applied this framework to sport by describing the parallel engagement of both ventral and dorsal systems that occurs, and, their relative contribution prior to and after the onset of movement. As such, a vast amount of experimental of research into interceptive tasks may have only engaged the ventral pathway (vision for perception) during video-based tasks, while others may have only addressed the dorsal pathway (vision for action) when utilizing ball machines that lack pre-ball flight information (Panchuk et al., 2013). For example, cricket batters have been shown to execute fundamentally different movement patterns, relying on different information–movement couplings, as a result of batting against a bowling machine instead of a bowler. This is also congruent with Travassos et al. (2013) meta-analysis findings that expertise advantages over novice performers are relative to the similarity between the behavior performed in a simulated setting, compared to the actual behavior in the performance environment. These findings highlight the need for some experimental analysis of skilful behaviors to occur in more representative, field based performance environments (Pinder et al., 2011b).

Previous experimental work on cricket batting actions have commonly utilized ball machines, explicit task instructions or a combination of both to investigate technical aspects of the movement (Stretch et al., 1998; Taliep et al., 2007; Weissensteiner et al., 2011). While this has revealed invaluable information regarding the incredible spatio-temporal abilities of skilled performers, the lack of realistic perceptual information and task goals impacts the ability of performers to execute realistic and adaptive actions. For example, batting strokes performed with a singular front foot movement has been the primary movement investigated. However, as demonstrated by Pinder et al. (2012), cricket batters execute strokes off both the front foot and back foot. It is also unclear the number of foot movements different skill level batters perform within more realistic settings. Explicit and narrow task goals given to the performer limit their ability to demonstrate their movement adaptability, therefore limiting our understanding of expertise. In realistic performance environments, movement behaviors are also perceived as either ‘functional’ or ‘dysfunctional’; dependent on whether they are deemed by the individual to meet the task goals within their environment (Davids et al., 2003). Task-goals that are purposely ambiguous and open ended, such as “score as many runs as possible without being dismissed,” can provide a unique opportunity to analyze the way in which different skill level performers address their movement functionality, and go about achieving the task goal.

From an ecological perspective, cognitions, emotions, and intentions play a powerful role in constraining perception and actions, which in turn are mutually constraining (Araújo et al., 2018). These processes are deeply integrated in terms of how they underpin performance. In the context of tests of expertise, intentions are important given they frame the interactions of batters with task and environmental constraints to facilitate changes between or refinement of different functional patterns of behaviors. Specifically, an individual’s intentions within a task, attention to various perceptual information and resulting adaptation of motor behaviors shape emergent skilful behaviors (Jacobs and Michaels, 2007). Araújo et al. (2005) investigated the decision-making strategies of sailors during a dynamic simulated regatta task, recording the actions and cognitions of elite and novice level sailors. They reported novice performers attended to their own individual movements (i.e., sailing maneuvres) more often than experts, who in turn attended to more adversarial informational variables (i.e., wind conditions). This is consistent with a large body of work that has highlighted the advantages of skilled performers focusing their attention externally, during both simple tasks (Wulf et al., 2001; McNevin et al., 2003) and more complex interceptive timing tasks (Castaneda and Gray, 2007). From an ecological dynamics perspective, this internal focus on individual movement components likely interferes with the self-organization process of performing an action. This difference in cognitive focus highlights a change in behavior that occurs at some point during skill development.

Despite this intertwined relationship between cognitions, emotions, perceptions and actions, few studies have considered cognitions and emotions of different skill level participants during tests of expertise, alongside their motor behaviors. One exception is in the Araújo et al. (2005) experiment discussed above, however, it is unclear, however, whether the individuals completing the task perceived their own performance as successful or unsuccessful, or what information within the performance environment sailors would base their assessment of ‘success’ upon. Given the aforementioned findings of Araújo and colleague’s, one can hypothesize that individuals likely perceive successful performance upon either performing optimal technical or process-focused movements (e.g., executing flawless sailing maneuvres), or capitalizing on the available opportunities for action, based on information in the environment (e.g., outcome-based). Understanding how individuals at various skill levels perceive their own successfulness at meeting task goals would provide more insight into the development of skilful cognitive behaviors. Finally, an individual’s emotional state plays a key role in influencing information–movement couplings. Certain emotions have been shown to influence the affordances (i.e., the opportunities for action provided by the environment; Fajen et al., 2009) perceived and acted upon by an individual when performing a task (Pijpers et al., 2006; Graydon et al., 2012). For example, climbers were tasked with climbing a wall during two conditions that caused either high or low levels of anxiety. During the high anxiety inducing condition, performers were found to underestimate their action capabilities (Graydon et al., 2012), executed more actions and demonstrated a narrower focus of attention when perceiving informational variables (Pijpers et al., 2006). Clearly, emotions play a vital role in performance and need to be considered as an interacting constraint influencing the production of functional movements. Recently, there have been calls for research to better address this relationship between action, cognition and emotions during learning experiences (Headrick et al., 2015b). It is argued that this approach can further our understanding of how individual learners interact with specific task demands and their environment, at different stages of their development.

The purpose of this study was to explore the interacting actions, cognitions and emotions produced by professional, amateur and junior level cricket batsman during a representative training scenario. Batters performance scores, actions (motor skills), cognitions (perceptions of self-performance and intentions) and a range of emotions were all recorded *in situ* to better understand the resultant emergent behavior. It was predicted that professional state level batters would outperform both amateur and junior batters, while amateur batters would outperform those junior batters, in all outcome measures and display more functional co-ordination measures (i.e., cricket specific actions). It was also predicted that those professional state level batters would perceive themselves to have ‘won’ more overs than their less skilled counterparts, demonstrate an external focus on outcome when evaluating prior performance and strategizing about how to score more runs for upcoming performance. In contrast, both amateur and junior batter’s cognitions, would be internally focused when thinking about their own prior and upcoming performance. Finally, professional state level batters would report different emotions with lower nervousness emotion ratings at the beginning of the scenario, and higher fulfillment ratings at its conclusion, than both amateur and junior batters.

## Materials and Methods

### Participants

Twenty-two cricket batters were invited to participate in this study. Six state level (age: M = 23.5 ± 3.8 years; height: M = 182.7 ± 5.5 cm; weight: 84.5 ± 3.2 kg), eight amateur senior grade club level (age: M ± SD = 25.4 ± 1.7 years; height: M ± SD = 179.5 ± 3.9 cm; weight: M ± SD = 76.8 ± 7.7 kg), and eight junior state representative batters (age: M ± SD = 14.2 ± 0.3 years; height: M ± SD = 171.5 ± 9.4 cm; weight: M ± SD = 62.0 ± 14.6 kg) were tested during their pre-season. Six amateur grade senior club level right arm pace bowlers (age: M = 24.2 ± 3.7 years; height: M = 180.8 ± 5.9 cm; weight: 74.3 ± 7.1 kg) were also recruited to bowl to all participants during testing. Each bowler was randomly assigned to deliver 11 of the 66 total overs to be bowled during the skills test. University ethics approval was obtained to conduct the study, and informed written consent was provided by all participants, including parental consent, prior to commencing the experiment.

### Procedures for the Batting Test

A representative cricket batting task was developed that would allow for skilful cricket batting behaviors, to be examined. Participants used their own bats and were required to wear standard protective equipment, which included helmet, gloves, thigh guards, leg pads, and abdominal protector. The training test scenario was designed to simulate the middle period of a limited overs game and consisted of batters receiving 18 balls from 2 to 3 bowlers. To create a more representative design, 8 fielders in the form of mannequins with cones placed 1.5 m either side to signal the horizontal area the represented fielder would hypothetically cover in this scenario were placed in standard fielding positions typical of the game scenario. Plastic poles (height = 2 m) were also placed on each cone to signal the vertical area the fielder could cover.

#### Scoring System

Participants were awarded ‘runs’ by hitting the ball into any one of the seven spaces between the fielders. Four runs were awarded if the ball traveled to the boundary (40 m from the batter’s crease), two runs if the ball traveled more than 20 m but did not reach the boundary, and one run if the ball traveled more than 10 m from the batter’s crease. If a ball was struck in the air to any one of the fielding positions, it was classified as ‘out’ (dismissal) and the batter was told they would lose 8 runs. This was included to encourage a risk versus reward scenario similar to a game (note: the batter did not actually lose 8 runs for every dismissal when analyzing results).

#### Bowlers

**FIGURE 1:**
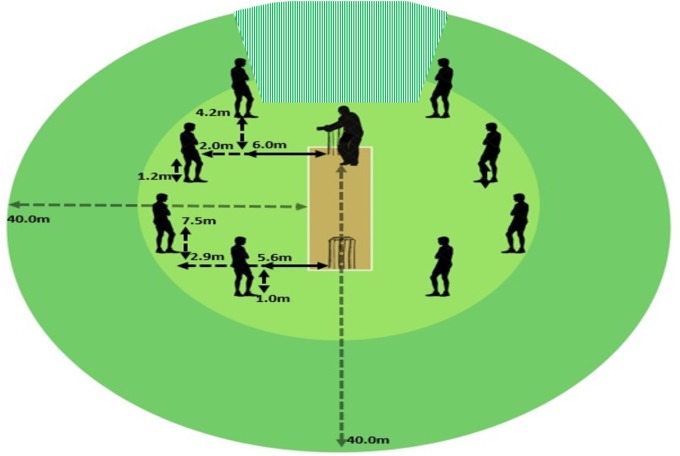
Testing setup depicting the position of each mannequin, which are mirrored on the other side of the field. Scoring zones exist in between mannequins (excluding shaded area behind the batter).

The state level and amateur senior batters faced right arm medium pace bowlers (approximately 115 km/h) bowling with pre-used Australian regulation balls (156 g; Kookaburra Turf Rejects). The junior representative players faced these same bowlers at a marginally reduced speed (approximately 100–105 km/h) for safety reasons. To achieve this, bowlers simply bowled off a shorter run-up. A radar gun (Stalker Radar Pro, Plano, TX, United States) was positioned in front of the umpire to monitor the bowler’s speed for each ball and ensure that the batters were experiencing bowling of similar speeds. Markers were placed every 2 m along the side of the pitch from the batter’s stumps to code ball length. A standardized field was set for all participants to visually represent the seven scoring opportunities available (Figure 1). In order to standardize the test for each batter, bowlers were given a randomized script of what lengths to bowl each over. In each over the bowler being asked to include four good length deliveries (ball pitching approximately 4 to 8 m from the batman’s stumps), one full of a length delivery (0 to 4 m from the stumps) and one short of a length delivery (over 8 m from the stumps, however, not bouncing above the batter’s head). To help guide the bowler, they were directed to use the cones placed either side of the pitch as a guide to length. Illegal deliveries (e.g., ball bouncing over the batter’s head or the ball traveling outside the wide lines) were not included and instead bowled again.

### The Test Protocol

Five minutes prior to commencing the test, each participant was read the following script describing the game scenario;

“You are the first batter coming in to bat after a wicket has just fallen on the last ball in the previous over. The game is at an even position for both teams, and your role is to score as many runs as possible without being dismissed. You are currently in the early to middle overs of a limited overs game and will receive 3 overs (18 balls). If you are “out” you will be deducted 8 runs from your final score.”

Each batter then completed a brief warm-up p facing 18 ‘throwdowns’ (over arm throwing at approximately 80 km-h s) from the three bowlers to familiarize them with the pitch (i.e., playing surface) conditions. The testing procedure involved batter’s facing 18 deliveries from the bowlers (in blocks of 6 balls labeled ‘overs’) with at least 3 min rest between each over. Batter’s then completed the test which took approximately 15 min.

### Data Collection

#### Actions

In order to record the skilful actions of batters, two high-speed cameras (Baslar, Baslar Ace acA2000, Germany; Casio Exilim, Japan) capturing at 300 frames per second were utilized. All camera distances were measured from the center of the batter’s crease. The front-on camera was placed on a hydraulic tripod and positioned directly in line with the batter (camera height = 5 m; distance from the center = 60 m). A second camera was positioned side-on at a 90-degree angle from where the batter was facing (camera height = 1.5 m; distance from center = 50 m; Figure 1). Video play back allowed the investigators to code each trial using subjective ratings including (1) quality of bat–ball contact (QoC) (Müller and Abernethy, 2008), (2) force of bat-swing (FOBS) (Mann et al., 2010), and (3) footwork technique rating (Table 1).

**Table 1:** Operational definitions of the three categories utilized in each subjective rating of quality of bat–ball contact, force of bat-swing, and footwork technique.

Rating	Quality of contact (QOC)	Force of bat-swing (FOBS)	Footwork technique
2	*Good contact* Ball contacts the bat face and travels in a direction consistent with the plane of the bat-swing.	*Complete swing* Complete follow-through of bat-swing after anticipated bat–ball contact.	*Deliberate movement* Transfers weight by stepping forward or backward, and foot is not in motion at time of contact.
1	*Poor contact* Ball contacts the edge of the bat or does not travel in a direction consistent with a plane of the bat-swing.	*Incomplete swing* Incomplete follow-through of bat-swing after anticipated bat–ball contact.	*Readjustment movement* Initially transfers weight forward or backward, however makes a readjustment movement in the final quarter of total ball flight time, prior to contact.
0	*No contact* Ball does not contact the bat when the batter attempts to play a shot.	*Defensive shot* No follow-through of bat-swing after anticipated bat–ball contact.	*Evasive movement* Does not transfer weight forward or backward, or jumps away from the line of the ball prior to contact.

#### Cognitions

In order to record the cognitions of batters during the test, a brief (on average each interview lasted 4 min) confrontational-style interview was conducted with the batter after each over. The purpose of the semi-structured, confrontation interview method was to gain a more in depth investigation of the cognitive states in relation to the behavior of the three skill level batters when engaged with an open-ended goal-orientated task. This process involved the participant reviewing their performance and being prompted to describe their thoughts and activity after every over (Gernigon et al., 2004; Poizat et al., 2012). Specifically, questions were designed to better understand the goals of batters. Batters were asked a series of open-ended questions centered upon how they judge and evaluate their own performance in relation to their own goals for each over. For example, the first two questions *“who do you think won that over?” and “why do you think this was the case?”* aimed to set up the rest of the interview questions. As the interview progressed, more purposeful question were asked, for example: *“What do you think the opposition’s plan was?”* or “*Why did you employ that strategy this over?”* or *“What influenced your game-plan that over?”* Importantly, these questions were designed to encourage participants to share and discuss scenarios that are evidenced of their thought processes during the scenario (Maxwell, 1992). Interviews were conducted immediately following each over (6 deliveries) allowing participants to show, simulate, relate and comment on their performance and underpinning thought processes (Hauw and Durand, 2004).

#### Emotions

The Sports Learning and Emotions questionnaire (SLEQ) was utilized just prior to the first ball being bowled, and again immediately following the last ball of the test. The results are presented as the total SLEQ score, and then separated into four factors which include enjoyment, nervousness, fulfillment and anger. The questionnaire asks participants to rate 17 words on a scale from 0 (not at all) to 4 (extremely). For example, the scores for ‘happy,’ ‘fun,’ ‘joy,’ ‘enjoyment,’ and ‘excited’ are all averaged to provide an overall score for the factor of enjoyment.

In order to record the emotions of batters, a SLEQ (Headrick et al., 2015a) was administered before the start of the 18 balls and immediately at the conclusion of the experiment. The questionnaire required participants to respond to a list of words that described an emotion (e.g., happy, frustrated, pressure, excited) by selecting a number between 0 (not at all) and 4 (extremely) that best represented how they currently felt.

#### Data Analysis

The combination of kinematic and performance outcome measures along with retrospective verbalizations of cognition and affective states (via the SLEQ questionnaire) provides an integrated analysis of the observable emergent behavior.

#### Actions

The bat–ball contact quality rating and FOBS rating both use a validated rating system that scores a 2, 1, or 0 points for each trial (Table 1). A third subjective rating was included, after viewing the trials, to address the various footwork coordination patterns employed by batters. A rating scale was developed in consultation with two experienced, elite coaches (one former international coach and one current international coach). It was designed to encompass three common movements employed by batters in this experiment. Points were assigned for movements that were perceived to be more efficient, based on coaching manuals and the perceptions of experienced elite coaches (e.g., Woolmer et al., 2008). For example, stepping or jumping away from the ball is thought to reduce the ability of a batter to contact the ball, or strike it powerfully. In contrast, a batter transitioning their bodyweight forward or backward to move into line with the ball prior to contacting the ball has been suggested as an effective way of powerfully striking the ball. Intraclass (0.79) and interclass (0.83) correlation coefficient demonstrated acceptable levels of reliability. Finally, batting characteristics included percentage of shots played by stepping ‘forward’ or ‘back’ (i.e., the direction of the last movement prior to bat–ball contact is either forward toward the ball or backing away from the ball), percentage of vertical or horizontal bat shots, and percentage of shots played along the ground or in the air.

#### Cognitions

In order to identify and interpret patterns of meaning within the qualitative data, recordings were transcribed, initial codes were generated, and a review for potential themes related to the perceptions of performance (Charmaz, 2006) was conducted by the lead author. Finally, as these concepts emerged they were discussed with two critical friends (i.e., the 3^rd^ author who had specific expertise in cricket batting and an independent qualitative researcher) in order to encourage reflection upon, and exploration of, multiple and alternate interpretations of the data (Smith and McGannon, 2018, p. 113). These themes were then titled and defined based on the broader concept they represented (Vaismoradi et al., 2016).

For the purpose of this study, and to address its aims, three key themes were coded for analysis. Firstly, based on their response to the question “who won the over, yourself or the bowler?” batter’s perceptions were coded as either a ‘win,’ ‘even,’ or a ‘loss’ The follow up question broadly asked “why do you think you won/it was even/lost?” elucidated five codes: (1) the ability (or lack of) to score runs; (2) being (or not being) dismissed; (3) (good or poor) execution of the batsman or (4) bowler; or (5) an emotional cause (e.g., felt/didn’t feel comfortable). The final question broadly asked, “what was your game plan that over?,” revealed six codes which included: (1) scoring runs by describing the process or outcome in which they would be scored; (2) limit the number of dismissals; (3) refer to making a technical change during the over; (4) achieving bat–ball contact; (5); or other, which is a combination of two codes that include “assess the conditions” (coded twice) and (6) “no plan” (coded once). Descriptive statistics of the relative number of times each code appeared per skill level group are presented in Section “Results.”

#### Emotions

The SLEQ was utilized just prior to the first ball being bowled, and again immediately following the last ball of the test. The results are presented as the total SLEQ score, and then separated into four factors which include enjoyment, nervousness, fulfillment, and anger. The questionnaire asks participants to rate 17 words on a scale from 0 (not at all) to 4 (extremely). For example, the scores for ‘happy,’ ‘fun,’ ‘joy,’ ‘enjoyment,’ and ‘excited’ are all averaged to provide an overall score for the factor of enjoyment. All five scores are presented as pre-test and post-test measurements for each of the three skill groups.

### Statistical Analysis

In order to analyze the actions of batters, including objective measures (runs scored, scoring shots and batting characteristics) and subjective ratings (QOC, FOBS, and footwork technique), separate one-way ANOVAs were conducted. Where further analysis of the different movements (readjustment movements vs. no readjustment movements) were required; in this instance, a two-way mixed ANOVA was conducted. *Post hoc* (Tukey) pairwise comparisons were then undertaken to determine which comparisons were statistically significant. Cognitions were presented using descriptive statistics. Two-way repeated measures mixed ANOVAs were used for the emotions data when comparing pre-test and post-test measurements across all skill levels. For all ANOVAs, the Greenhouse–Geisser correction was applied for any violations of Maulchy’s test of sphericity. *P*-value was set to 0.05 level of significance.

## Results

Analysis of the bowler’s deliveries was initially undertaken to check that each batter received the ‘same’ test. Analysis revealed no difference between the different skill level batters, in terms of the three different lengths bowled, which included full of a length deliveries *F*(2,19) = 0.06, *p* = 0.94, η^2^ = 0.06 (state: 4.50 ± 2.07; amateur: 4.63 ± 1.30; junior: 4.38 ± 0.92), good length deliveries *F*(2,19) = 0.57, *p* = 0.57, η^2^ = 0.06 (state: 9.00 ± 2.37; amateur: 9.75 ± 1.17; junior: 9.87 ± 0.71) and short of a length deliveries *F*(2,19) = 1.89, *p* = 0.18, η^2^ = 0.17 (state: 4.50 ± 1.05; amateur: 3.63 ± 0.74; junior: 3.75 ± 0.89).

### Performance Outcomes

**FIGURE 2:**
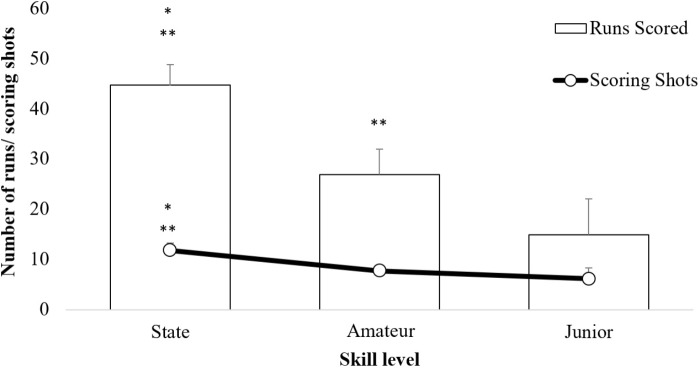
Average number of runs scored and scoring shots played by state, amateur and junior level batters. ^∗^Significantly different from amateur level batters (*p* < 0.05); ^∗∗^significantly different from junior batters. Error bars represent standard deviation.

Significant differences were found between skill levels for runs scored *F*(2,19) = 46.15, *p* < 0.05, η^2^ = 0.83 and the number of scoring shots played *F*(2,19) = 23.17, *p* < 0.05, η^2^ = 0.71. *Post hoc* tests revealed state level batters scored significantly more runs (44.67 ± 4.08) and played more scoring shots (11.83 ± 1.47) than amateur level batters (26.88 ± 5.06; 7.75 ± 0.46) and junior batters (14.88 ± 7.22; 6.13 ± 2.23). Likewise, amateur level batters scored significantly more runs than junior level batters, however, no difference was found between the number of scoring shots played between these two groups (Figure 2).

### Technical Factors

**FIGURE 3:**
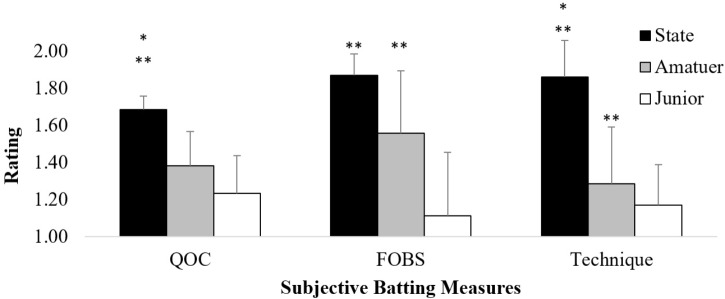
Average quality of bat–ball contact (QOC), force of bat-swing (FOBS) and technique rating. ^∗^Significantly different from amateur batters (*p* < 0.05); ^∗∗^significantly different from junior batters. Error bars represent standard deviation.

Analysis of technical factors revealed significant effects for QoC *F*(2,19) = 11.94, *p* < 0.05, η^2^ = 0.56, FOBS *F*(2,19) = 11.57, *p* < 0.05, η^2^ = 0.55, and footwork technique ratings *F*(2,19) = 14.29, *p* < 0.05, η^2^ = 0.60. *Post hoc* tests revealed state batters had significantly better QoC (1.68 ± 0.07) than both amateur (1.38 ± 0.19) and junior batters (1.23 ± 0.21). Both state (1.87 ± 0.11) and amateur batters (1.56 ± 0.34) also had significantly greater FOBS than junior level batters (1.11 ± 0.34). Finally, state batters demonstrated higher technique ratings (1.86 ± 0.20) than both amateur (1.28 ± 0.31) and junior batters (1.17 ± 0.22), while amateur batters also rated significantly higher than junior batters (Figure 3).

### Batting Strokes

**FIGURE 4:**
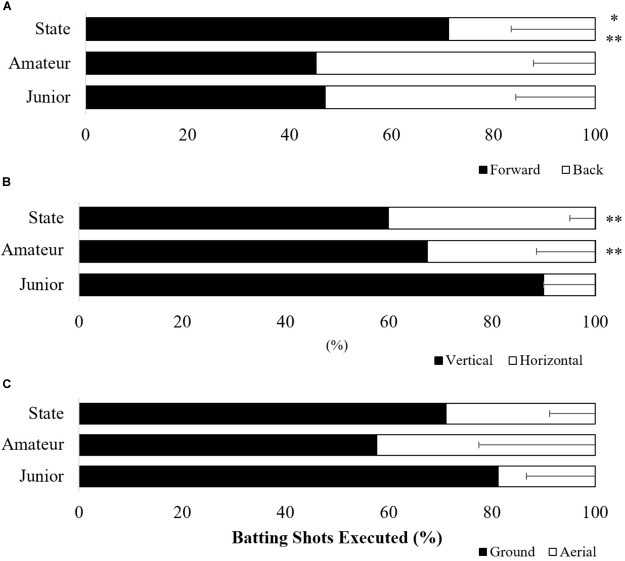
Ratio of shots played off the front and back foot **(A)**, shots played with a vertical or horizontal bat **(B)**, and shots played along the ground or in the air **(C)**. ^∗^Significantly different from amateur batters (*p* < 0.05); ^∗∗^significantly different from junior batters. Error bars represent standard deviation.

In regards to the way in which batters executed their strokes, there was a significant difference in the percentage of shots executed off the front foot or back foot *F*(2,19) = 6.45, *p* < 0.05, η^2^ = 0.40 and percentage of vertical or horizontal bat shots *F*(2,19) = 10.52, *p* < 0.05, η^2^ = 0.53. No difference was found for shots played along the ground or in the air *F*(2,19) = 13.58, *p* = 0.10, η^2^ = 0.22. Figure 4A shows state batters played significantly more shots off the front foot (71.26 ± 16.49%) compared to amateur (45.19 ± 12.16%) and junior batters (47.05 ± 15.55%). Junior batters were also found to play significantly more vertical bat shots (90.07 ± 10.0%) than both state level (60.00 ± 4.86%) and amateur level batters (67.50 ± 11.39%; Figure 4C).

### Movement Characteristics

There was a significant difference in the average number of movements *F*(2,19) = 11.52, *p* < 0.05, η^2^ = 0.55 executed by the batters of different skill levels. *Post hoc* tests revealed that state level batters performed significantly less movements than both amateur and junior level batters. Similarly, there was a significant difference in percentage of trials executed with a secondary movement *F*(2,19) = 3.90, *p* < 0.05, η^2^ = 0.29 and a readjustment movement *F*(2,19) = 25.32, *p* < 0.05, η^2^ = 0.73 between skill levels. *Post hoc* tests revealed state level batters performed significantly less secondary movements than junior batters, and performed significantly less readjustment movements than both amateur and junior level batters.

**FIGURE 5:**
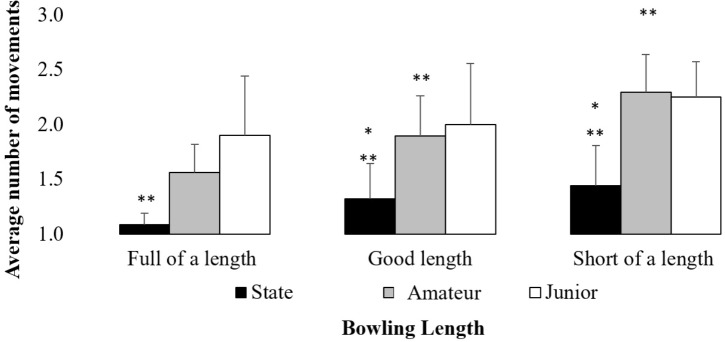
Comparison of the number of executed movements by state, amateur and junior batters when facing different length deliveries. ^∗^Significantly different from amateur batters (*p* < 0.05); ^∗∗^significantly different from junior batters.

Further analysis of batter’s movements revealed that, regardless of ball length, state level batters moved significantly less than their less skilled counterparts. Differences in the number of movements executed were reported for full of a length deliveries *F*(2,19) = 10.69, *p* < 0.05, η^2^ = 0.73, good length deliveries *F*(2,19) = 8.92, *p* < 0.05, η^2^ = 0.48 and short of a length deliveries *F*(2,19) = 6.47, *p* < 0.05, η^2^ = 0.41. *Post hoc* tests revealed state batters executed significantly less movements compared to junior batters when facing a full of a length delivery, and significantly less movements than both amateur and junior batters when facing good length and short of a length deliveries (Figure 5).

**FIGURE 6:**
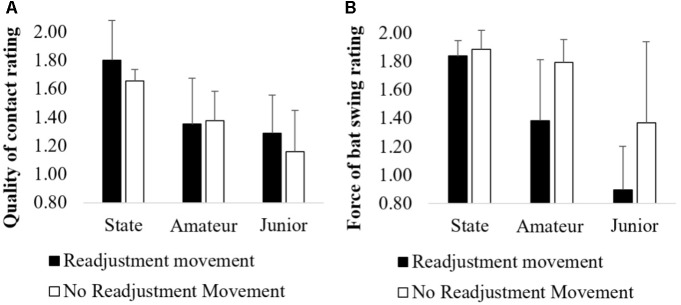
Comparison of the **(A)** QoC and **(B)** FOBS during trials where batters executed a readjustment movement.

There was no significant interaction between movement type (i.e., readjustment or no readjustment movement) and skill level for the QoC rating *F*(2,36) = 0.49, *p* = 0.62, η^2^ = 0.03. Therefore, an analysis of the main effect for movement type was performed, which similarly indicated no significant main effect *F*(1,36) = 1.01, *p* = 0.32, η^2^ = 0.03 (Figure 6A). There was also no significant interaction between movement type and skill level for the FOBS rating *F*(2,36) = 1.16, *p* = 0.33, η^2^ = 0.06. However, there was significant main effect for movement type *F*(1,36) = 7.54, *p* < 0.05, η^2^ = 0.17 (Figure 6B). Executing a readjustment movement (1.28 ± 0.48) resulted in batters having a lower FOBS rating when compared to trials where batters did not execute a readjustment movement (1.67 ± 0.42).

### Cognitions

When batters were asked at the end of each over who they believed had won, i.e., themselves or the bowler, all groups exhibited similar responses; state (win 33.3%; balanced 6.7%; loss 60.0%), amateur (win 18.2%; balanced 27.3%; loss 54.5%) and junior batters (win 29.7%; balanced 12.5%; loss 58.3%) with all groups evaluated losses as occuring more often than both wins or balanced contests.

When batter’s were asked to explain why they concluded that they had won, lost or drew the over, the majority of the state batter’s responses related to outcome-goals (Figure 4). That is, their ability to score runs (30.4%) or whether or not they were dismissed (47.8%). Their percieved execution of shots (17.4%) or emotions (e.g., felt/ didn’t feel confident; 4.4%) were less prominent factors. Amateur batters expressed similar response rates in regards to scoring ability (40.7%) and whether they were dismissed (37.0%), as being dominant factors. The execution of both the batter (11.1%) and opposition bowler (11.1%) were the only other factors mentioned in their responses. Finally, junior batters highest response was their own ability to execute (48.3%), while their percieved ability to score (17.2%), dismissals (13.8%), emotions (17.2%), and execution of the opposition bowler (3.5%) were regarded less when justifying their perceptions of winning or losing the over. Interestingly, the bowler’s execution was never mentioned as a contrubuting factor by any batter.

When prompted to describe their game-plans during each over, both amateur and junior batters expressed far more diverse responses compared to state batters. Scoring runs (87.5%) was the highest response rate for state batters, with making technical changes (12.5%). Amateur batters included scoring runs (57.7%) and achieving bat–ball contact (26.9%) as the predominant goals in their game-plan, while limiting dismissals (11.5%) and other/none (3.9%) were briefly mentioned. Junior batter’s reponses were the most varied, with scoring runs (50%) and making technical changes (25%) being the most predominant responses, followed by achieving bat–ball contact (10.7%), other/none (10.7%), and limiting dismissals (3.6%).

### Emotions

There was no significant interaction between skill level and time for enjoyment *F*(2,19) = 1.10, *p* = 0.35, η^2^ = 0.10; fulfillment *F*(2,19) = 0.64, *p* = 0.54, η^2^ = 0.06, anger *F*(2,19) = 1.66, *p* = 0.22, η^2^ = 0.15 or total emotions score *F*(2,19) = 1.48, *p* = 0.25, η^2^ = 0.13. Follow up main effects were not reported as they were not of direct interest to the aims of this experiment. There was, however, a significant interaction for nervousness scores *F*(2,19) = 9.07, *p* = <0.05, η^2^ = 0.49. Follow-up tests revealed differences between skill level *F*(1,7) = 22.77, *p* < 0.05, η^2^ = 0.77 and also between time *F*(2,19) = 8.13, *p* < 0.05, η^2^ = 0.46. During the pre-test, junior batters were found to have had significantly higher nervousness ratings than both state and amateur level batters. Similarly, junior batters also had significantly higher nervousness rating during their pre-test when compared to their post-test.

## Discussion

The purpose of this study was to explore the interacting actions, cognitions, and emotions produced by professional, amateur and junior level cricket batsman during a representative training scenario. The major findings revealed differences in cognitions, emotions, and actions and specifically that state level batters, when facing bowlers during a game-like scenario, (1) demonstrate superior technical actions (e.g., bat–ball contact, footwork); (2) exhibit different movement strategies, including executing more varied vertical and horizontal bat shots while on the front foot, and later initiation of their initial foot movement and downswing of the bat; (3) cognitively evaluated their performance based on different goal-orientations, i.e., outcomes (i.e., runs scored and whether a dismissal occurred), while formulating a strategy predominately centered on how they could score runs; and (4) reported lower nervousness levels than junior level batters.

### Actions

As expected, State level batters, when compared with both senior amateur and junior batters, scored more runs, played more scoring shots, and demonstrated higher rated QoC, FOBS, and footwork technique. These superior batting skill results are similar to those reported in the limited number of studies comparing different skill levels in cricket batting (Müller et al., 2006; Weissensteiner et al., 2011). However, a point of difference between this experiment and previously reported findings is the representative nature of the environment and the task demands that the participants sought to satisfy in the current study. Therefore, it is argued that the findings of this experiment can be generalized to the performance environment in which these behaviors occur. Specifically, the differences in both movement and cognitive behavior between different skill level batters occur as a result of the dynamic task constraint interacting with the cognitions of the batter (i.e., rules of the scenario) and the opposition bowler. As opposed to being tasked with executing a single coordination pattern (e.g., a forward defensive shot) against a ball projection machine or a pre-determined ball trajectory, participants were required to play a range of shots commensurate with the balance of risk and rewards inherent within a simulated game scenario. State level batters were able to demonstrate an expertise advantage in this task through superior temporal and spatial coordination that resulted in effective execution.

Interestingly, the emergence of a wide range of varied batting strokes (e.g., horizontal and vertical) and more functional foot work (e.g., less readjustment movements and greater technical footwork rating) was only displayed by state level batters. In contrast, lesser skilled batters demonstrated a lower range of options in their batting strokes and more varied, but less functional, foot work (Figures 3, 4A). Reinforcing the work of Stretch et al. (2000), the findings of this study demonstrate that more skilful batters are better able to perceive and act upon the affordances presented to them within their performance environment. That is, skilful batters in this experiment had more scoring shots available to them due to their superior technical coordinative ability coupled with an attunement to the multiple affordances available within the performance environment.

Examining the technical coordinative patterns of batters revealed those at state level executed less foot movements compared to amateur and junior batters, with less skilled batters executing more ‘secondary’ and ‘readjustment movements’ (Table 2). Further analysis revealed state batters executed less foot movements to full length deliveries than junior batters, while also executing less foot movements during good length and short of a length deliveries than both amateur and junior batters (Figure 5). In order to more clearly understand the link between number of foot movement phases and performance outcomes, the QOC and FOBS measures were compared for trials where a readjustment movement occurred and trials where it was not (Figure 6). While there was no difference between the QoC and trials where a readjustment movement was performed, regardless of skill level, force of bat-swing ratings were lower in trials where batters performed a readjustment movement. This finding suggests that readjustment movements may be a functional movement solution for batters whose primary goal is to simply achieve bat–ball contact, but dysfunctional for a task-goal that requires the batter hit the ball with relative force to score runs. This emergent behavior is unlike the movements presented in previous cricket studies (Pinder et al., 2011a; Weissensteiner et al., 2011) where batters are reported as only having one movement phase, or their movement timings are only reported as the initiation and cessation of their first and last movement respectively.

**Table 2:** Average number of foot movements and percentage of trials where batters performed an initial movement, a secondary movement, or a readjustment movement.

	Average number of movements	Initial movements	Secondary movements	Readjustment movements
State	1.30 ± 0.21% ^∗ ∗∗^	100.0 ± 0.0%	12.15 ± 13.19%^∗∗^	18.63 ± 12.76% ^∗ ∗∗^
Amateur	1.87 ± 0.33%	100.0 ± 0.0%	35.31 ± 24.07%	51.52 ± 15.73%
Junior	2.04 ± 0.30%	100.0 ± 0.0%	46.65 ± 23.86%	58.20 ± 10.21%

*^∗^Significantly different from amateur batters (p < 0.05); ^∗∗^significantly different from junior batters.*

### Cognitions

**FIGURE 7:**
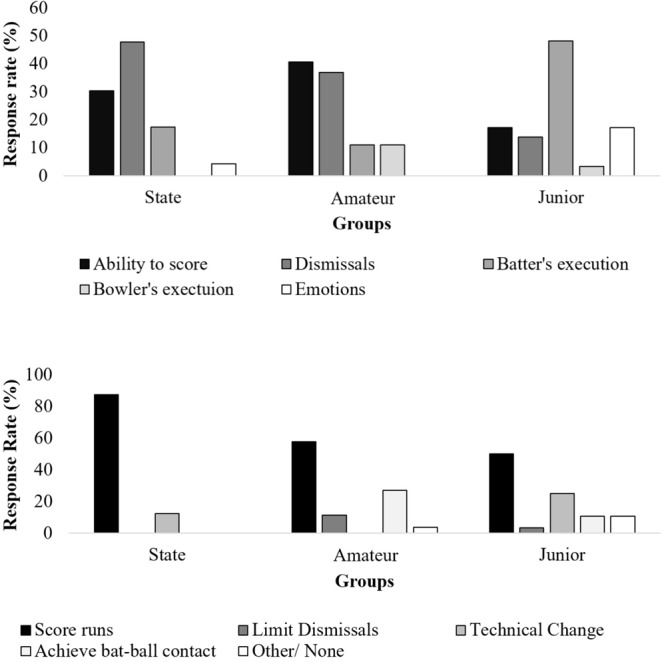
Frequency distribution of state, amateur and junior cricketer’s responses when asked why they perceive that the over was won, lost, or balanced.

Skill level differences in cognitions were found in the type of game-specific information batters utilized to evaluate performance and strategize. While state level batters scored significantly more runs than both amateur and junior batters, and no difference was found in the number of dismissals, all groups reported similar percentage of overs they perceived to have ‘lost’ to the opposition bowler (Figure 7). This finding is crucial to the interpretation of the following cognition data and highlight that the differences found in cognition cannot solely be explained by state level batters being more successful during this task than their lesser skilled counter parts. Both state, amateur and junior level batters all reported similar perceptions of overs they perceived to have lost during the scenario. Therefore, while state batters did score more runs and demonstrate more efficient motor behaviors, their perceptions of success were no different to the perceptions of amateur and junior level batters and highlight the goals of the batters shaped their cognitive evaluations of the outcomes. Understanding the dynamic cognitions of batters is important as intentions play a significant role in shaping perception and action (Araújo et al., 2018). The following differences in cognition at least partially represent the way in which different skill level batters regulate their cognitions during a game-scenario.

As hypothesized, both state and amateur level batters remarked that the ability to score runs, and whether they were dismissed, were key factors when evaluating their performance (Table 3), however, junior batters were far less concerned with these game-specific outcomes. Instead, how well or how poorly they executed during the over was the prevailing factor when evaluating their performance (40.7%). That is, junior batters were more likely to respond with comments about their own ability to achieve bat–ball contact or be in an effective position when contacting the ball. One possible reason for this finding may be the coaching practices to which less skilled players are typically exposed. For example, Roberts (2011) analysis of cricket coaching practices found that the coaches largely provided technical feedback through direct instruction (e.g., *I can see it is going wrong but I only see the technique. That is what I want to correct...*”; p. 42). A focus of technical feedback from coaches can be harmful as direct instruction on technical movements shifts an individual’s focus internally, disrupting automized movements, and leading to poorer skill execution (Wulf et al., 1998). Additionally, an internal focus has the potential to interfere with the search for, and attunement toward, the multiple affordances in the performance environment (Handford et al., 1997). A narrow and internalized focus has also been previously reported during experiments where participants are subjected to anxiety provoking conditions (Pijpers et al., 2005), however, this will be discussed further in a later section.

**Table 3:** Frequency distribution of state, amateur and junior cricketer’s responses regarding their game plan each over.

	Score runs	Limit dismissals	Technical change	Achieve bat–ball contact	Other/none
State	87.5%	0.0%	12.5%	0.0%	0.0%
Amateur	57.7%	11.5%	0.0%	26.9%	3.9%
Junior	50.0%	3.6%	25.0%	10.7%	10.7%

When batters were asked to verbalize their game-plan after the over, those at state level made responses that overwhelmingly referred to scoring runs in specific areas (e.g., *“Try to hit as straight as I can, and then the short [length delivery] one, just pick that gap there [referenced particular gap between two fielders]”)* or using a specific strategy to score (e.g., *“The game plan is there when the ball is there. I’m kind of playing see it, hit it. But I’m looking, cause [sic] the biggest gap is straight, so if the ball is straight I’m looking to hit it straight”).* In contrast, amateur batters additionally referred to achieving bat–ball contact (e.g., *“Just hit the ball”; and “Similar to the first 6 balls, still pushing for anything full to try and hit a little harder. And anything short, just try and get a bat on it if possible”*). Junior batters additionally referred to having to make technical changes (e.g., *“…I would have liked to have played off the front foot more and played some shots much straighter”; and “Yeah to move my feet and try get it [the ball] through the offside”*). Similar to the findings of Araújo et al. (2005) during their investigation of sailors, skilled batters verbalized more regularly the opportunities for action available relative to their performance environment, while those lesser skilled explored their own motor behavior and reflected how their movements could be more functional.

### Emotions

The ability to regulate emotions was another factor that distinguished between different skill level batters. Perhaps not surprising given that the bowlers were ‘below’ their current performance level and may not be perceived as a threat to their goals. State level players exhibited less nervousness prior to performance than their junior level counter-parts. In contrast, junior players were facing bowlers who were ‘above’ their level and therefore may have been perceived as a great threat to success (Table 4). High levels of nervousness have been attributed to causing a narrowing of an individual’s focus of attention, similar to that of a novice performer (Masters, 1992; Pijpers et al., 2005). This, in turn, is thought to limit the affordances perceived and acted upon by individuals, such as making them more conservative in their actions (Pijpers et al., 2006). It is suggested that this cognitive-emotions relationship is a likely contributing factor to the internalized and narrower self-evaluative cognitions exhibited by junior batters. It is important to note that after this experiment, junior level batters reported lower levels of nervousness compared to a pre-test questionnaire. It is unclear whether this reduction is due to batters becoming more accustomed to the task, or that the task itself had finished. Future research is needed to examine *in situ* whether regulating emotions (as well as characterizing which emotions specifically) during performance is a distinguishing factor between skill levels in cricket batting.

**Table 4:** Sports Learning and Emotion Questionnaire (SLEQ) responses from state, amateur and junior cricket batters.

		Enjoyment	Nervousness	Fulfillment	Anger	Total score
State	Pre	2.28 ± 0.59	0.55 ± 0.41^∗∗^	2.07 ± 0.77	0.40 ± 0.59	5.30 ± 1.57
	Post	2.36 ± 0.70	0.40 ± 0.29	1.77 ± 1.03	1.05 ± 0.92	5.58 ± 1.49
Amateur	Pre	2.83 ± 0.73	0.81 ± 0.56^∗∗^	2.13 ± 0.78	0.63 ± 0.79	6.39 ± 1.76
	Post	2.85 ± 0.58	0.72 ± 0.51	2.10 ± 0.80	1.25 ± 0.97	6.92 ± 0.84
Junior	Pre	3.15 ± 0.61	1.94 ± 0.95^†^	2.85 ± 0.72	0.46 ± 0.46	8.40 ± 1.26
	Post	3.53 ± 0.51	0.47 ± 0.54	3.15 ± 0.28	0.42 ± 0.53	7.56 ± 0.39

*^∗^Significantly different from amateur batters (p < 0.05); ^∗∗^significantly different from junior batters; ^†^ significantly different from post-test.*

The primary limitations of this study included the logistical difficulty of standardizing bowlers to accurately bowl to their scripted lengths, the sample size of participants, and certain factors that relate to the representative design of the task. For example, fielders were substituted for mannequins and at no point changed positions in the field, in response to the batter’s scoring. This inherently created a more static performance environment. Batters therefore were tasked with striking the ball into any consistently present gap in the field, as opposed to striking it into a gap between fielders who could move and intercept the ball; thus changing the affordances present for the batter. Secondly, a clear purpose of this experiment was to go beyond earlier studies of batting technique that used bowling machines by utilizing real bowlers to capture true representations of batting performance (cf. van der Kamp et al., 2008). This goal presented some challenges in ensuring that each batter received the ‘same’ test. However, in line with Brunswikian theory, the more representative nature of the test is not so much a restrictive issue given the vicarious functioning of human behavior (Dhami et al., 2004). Therefore, it is proposed that the advantages far outweigh the limitations. The actions, cognitions, and emotions reported as batters interacted with bowlers to achieve their task goals during this study could not have been replicated by facing a ball machine. It is also evident from the results of this study that, by removing ball projection machines and replacing them with bowlers as a ball delivery method, it enabled batters to demonstrate an ability to perform multiple different co-ordination patterns in contrast to previous studies. A final limitation of the study, was the capacity to demonstrate how cognitions and emotions intertwine dynamically with ongoing perception and actions. Future studies should therefore address this challenge by capturing the impact of cognitions and emotions at key intervals throughout tests. Cricket batting is an ideal task vehicle for such studies as there are natural breaks in ‘action’ at the end of every over.

## Conclusion

A distinction between this study, and those previously conducted with cricket batters, is the attempt to maintain the dynamic relationship inherent between a batter and bowler by creating a representative test. State level batters demonstrated superior technical proficiency and an ability to score runs, which was reflected in their cognitive evaluations and strategizing that referenced how they went about scoring runs. Conversely, junior batters with their less proficient technical batting skills, in turn, exhibited cognitions directed toward achieving better skill execution. Interestingly, there was no significant difference in the number of scoring shots played by amateur and junior batters, yet amateur batter’s cognitions predominately referenced scoring runs when evaluating performance and strategizing. It’s suggested that junior batter’s higher level of nervousness further reinforced a narrow and internalized (cognitive) focus toward their own movements. The practical implications of this study stress the importance of viewing skill learning as more than a mastery of coordinative movements. A key element to skilful performance is the ability to adapt one’s actions and cognitive strategies to suit the performance environment, and manage the emotions that concurrently occur.

## Ethics Statement

This study was carried out in accordance with the recommendations of NHMRC, Victoria ethics committee. The protocol was approved by the Victoria University ethics committee. All subjects gave written informed consent in accordance with the Declaration of Helsinki.

## Author Contributions

JC was responsible for the data collection, analysis, and write up of the experiment. IR assisted with some of the data collection. Both IR and DF provided extensive feedback regarding the conceptualization of the experiment, appropriate statistical analyses, and write-up.

## Conflict of Interest Statement

The authors declare that the research was conducted in the absence of any commercial or financial relationships that could be construed as a potential conflict of interest.
